# Unraveling the Impact of Long-Term Rice Monoculture Practice on Soil Fertility in a Rice-Planting Meadow Soil: A Perspective from Microbial Biomass and Carbon Metabolic Rate

**DOI:** 10.3390/microorganisms10112153

**Published:** 2022-10-30

**Authors:** Zhanxi Wei, Hao Wang, Chao Ma, Shuyuan Li, Haimiao Wu, Kaini Yuan, Xiangyuan Meng, Zefeng Song, Xiaofeng Fang, Zhirui Zhao

**Affiliations:** 1 Qinghai 906 Engineering Survey and Design Institute, Xining 810007, China; 2 Qinghai Geological Environment Protection and Disaster Prevention Engineering Technology Research Center, Xining 810007, China; 3 Hebei Province Key Laboratory of Sustained Utilization & Development of Water Recourse, School of Water Resources and Environment, Hebei GEO University, Shijiazhuang 050031, China; 4 Research Center for Eco-Environmental Sciences, Chinese Academy of Sciences, Beijing 100085, China

**Keywords:** microbial biomass, carbon metabolism, meadow soil, phospholipid fatty acids, tillage, fertilization

## Abstract

Global agricultural intensification leads to a decline in soil quality; however, the extent to which long-term rice cultivation adversely impacts soil, based on chemical and microbial perspectives, remains unclear. The present study was conducted on a seed multiplication farm in Wuchang, Heilongjiang Province, China, to quantify changes in the nutrient properties and microbial profiles of meadow soil in cultivated (rhizosphere and bulk soil) and uncultivated paddy plots from spring to winter. A non-parametric method was used to compare carbon metabolism characteristics among the three groups of soil samples. Principal component analysis was used to distinguish soil chemical properties and carbon source utilization profiles among the soil samples across different seasons. Under rice cultivation, pH, organic matter, total nitrogen, and alkali-hydrolyzed nitrogen concentrations were generally higher in rhizosphere soils than in bulk or uncultivated soils. However, microbial biomass in cultivated soils was consistently lower than in uncultivated soils. There was a discernible difference in carbon substrate preference between summer and other seasons in the three sample groups. In conclusion, agricultural activities in rice cultivation could reshape soil microbial communities in the long term. Notably, specific cultivation activity may induce distinct soil microbial responses, which are more sensitive than chemical responses.

## 1. Introduction

In the wake of global environmental change, anthropogenic activities in different forms and degrees are constantly transforming land-use patterns [1]. The most typical examples are converting natural forests or pastures to farmland over large areas [1,2]. Land-use strategies prominently impact soil health [3,4], demonstrating the soil ecosystem’s continued capacity to sustain plants, animals, and humans [5]. As soil quality indicators, soil physicochemical and microbial properties have attracted considerable attention [6,7]. For example, changes in soil pH, organic matter, and total nitrogen (N; TN) contents, as well as microbial biomass (MB) and metabolic profiles, are frequently monitored to assess the impacts of land-use type on soil quality [8,9].

Owing to growing demands for food and bioenergy, many countries globally need to continuously increase their arable land area and agricultural productivity [1,10]. Consequently, intensive cultivation practices adversely influence soil physicochemical properties, inhibit local soil microbial activities, and impair soil function [4,8,11]. Tillage operations disturb the soil structure, alter soil physical properties (e.g., bulk density and moisture content), and reduce soil N, organic carbon (C) contents, MB, and enzyme activity [6,12]. As a crucial nutrient for sustaining the life processes of plants, P availability is influenced by several factors, such as pH and soil organic matter content, and eventually determines crop production [13,14]. However, irrational fertilization practices may lead to nutrient imbalance and shifts in microbial community structure [6,11]. For example, a high rate of N fertilization decreases the contents of extractable soil nutrients (e.g., phosphorus [P], potassium [K], and calcium) and the relative abundance of mycorrhizal fungi markedly, impairing agricultural production sustainability [6,13].

Rice, one of the most widely consumed cereal crops globally, is the staple food for more than half the global population [15]. Rice monoculture is a cropping strategy adopted worldwide, and double- or triple-sequential cropping is implemented in Asia’s lowland tropics and subtropics [7]. However, the sustainability of continuous monoculture systems is a source of concern [16]. Specifically, monoculture practices can increase soil compaction, reduce soil nutrient availability, and decrease soil physicochemical quality [16,17]. It has been proved that extractable nutrients (e.g., N and P) determine crop production; hence, interrupting nutrient accessibility threatens agriculture sustainability [18]. In addition, such practices decrease the abundance and diversity of soil microbes and reshape microbial community structure, altering soil microbiological quality [7,19].

Microbes play an essential role in governing the fertility of rice soils and plant uptake of nutrients [5,20]. Microorganisms inhabiting the rice rhizosphere are much more abundant than bulk soil [21]. Bacteria, fungi, and protozoans can accelerate nutrient mineralization, enhance nutrient availability, and supply nitrogen actively, thus promoting rice growth directly [22]. On the other hand, microbial biomass, community structure, and metabolic potential are common indicators reflecting the soil quality and stability [20]; degraded soil microbial communities may disrupt nutrient cycling, reduce organic matter decomposition, and thus limit rice yields [23]. Moreover, intensive rice monocultivation increases the risks of infection by diseases and pest infestation, in addition to facilitating the accumulation of soil-borne pathogens [24]. Therefore, soil microbial community manipulation may ensure the sustainability of paddy cultivation [22].

China is a leader in global rice production and consumes more rice than any other country [25]. Northeastern China is one of the six first-grade rice-growing regions in China [26], and the traditional high-quality rice planting area comprises Heilongjiang, Jilin, and parts of Liaoning and Inner Mongolia [27]. In particular, Wuchang rice (*Oryza sativa* L. ssp. *japonica*), a protected geographical indication product in China, has been planted in the meadow soil of Wuchang City in Heilongjiang Province for over 200 years [28]. Due to the low winter temperature and short growing season in Wuchang City, southern Heilongjiang, rice monoculture with winter fallow is the predominant cultivation system [27]. The meadow soil is expected to degrade following long-term—more than 200 years—rice cultivation.

The present study investigated the impacts of rice cultivation on soil quality in a seed multiplication farm in Wuchang City managed based on local cropping strategies, which is of great significance to improve the soil ecological environment of meadow soil and provides valuable data for the restoration and rational utilization of meadow soil ecosystem. The pH, nutrient properties (soil organic matter [SOM]), TN, alkali-hydrolyzed N [AHN]), and microbial profiles (MB and C metabolism characteristics [CMC]) of cultivated meadow soils were measured. All soil variables were compared with those in uncultivated soils, and their seasonal variation was observed. We hypothesized that: (1) rice cultivation practices negatively impact nutrient-related soil properties; (2) MB is reduced under rice cultivation, with higher values in the rhizosphere than in the bulk soil; and (3) CMC varies with the cultivation status and seasonal dynamics.

## 2. Materials and Methods

### 2.1. Study Area and Experimental Design

The field study was carried out in the No. 1 seed multiplication farm of Wuchang Agricultural Technology Extension Center (44°53′59″ N, 127°06′08″ E), Heilongjiang Province, China. The study area has a temperate continental climate, with a frost-free period of 124 days. The mean annual temperature and precipitation are 3.5 °C and 625 mm, respectively. The soil type on the farm is meadow paddy soil (Hapli-Stagnic Anthrosols) [29].

The experimental field was divided into two plots (1/3 ha per plot) based on their cultivation status. Plot I (uncultivated) had been abandoned for over 20 years, whereas Plot II (cultivated) had been grown with Wuchang rice for three consecutive years. The growing period of Wuchang rice generally starts in mid-May and ends in early October [30]. Every May, three chemical fertilizers were applied in plot II before plowing (0–20 cm depth). Specifically, the rates of application of N, phosphate (P_2_O_5_), and potash (KCl) fertilizer were 97.5 kg, 48.0 kg, and 90.0 kg per ha, respectively.

### 2.2. Soil Sampling and Preparation

Soil samples were collected from the 0–20 cm depth at four rice growth stages in 2019: transplanting stage (18 May, spring), maturity stage (18 August, summer), harvesting stage (15 September, autumn), and fallow stage (30 October, winter). Uncultivated soils (US) were collected from Plot I, whereas rhizosphere (RS) and bulk soils (BS) were sampled from Plot II. Each composite soil sample was obtained by combining five subsamples collected using a five-point sampling strategy. The fresh soil samples were sealed in a plastic bag, kept in an icebox, and then transported to the laboratory for subsequent analyses.

The collected soil samples were passed through a 2 mm stainless steel sieve to remove stones, plant roots, and other debris. Afterward, each sample was divided evenly into two parts: one part was employed in soil chemical analysis, and the other part was used to profile soil microbial community characteristics.

### 2.3. Soil Chemical and Microbial Analyses

After freeze-drying (Scientz-10N; Xinzhi Biotechnology Co., Ltd., Ningbo, China), soil pH, SOM, TN, and AHN were determined according to the standard methods published by Lu [31]. Soil MB and CMC were profiled using phospholipid fatty acid (PLFA) and Biolog EcoPlate analyses, respectively.

(1) *PLFA analysis*. PLFAs, a class of membrane-bound substances, have been commonly used to depict the soil microbial commnity structure and characterize the biomass of living microorganisms [12,32]. Approximately 6 g (accurate to 0.01 g) of freeze-dried soil samples were used to extract PLFAs based on a modified Bligh–Dyer method [33], which involved concentration/extraction/concentration/methylation/purification procedures, followed by air-drying [34]. Nonadecanoic acid methyl ester (33 μg/mL, CAS 1731-94-8, N5377-5G, Sigma, US, Chromatographically pure) was used as the internal standard. Two mL of *n*-hexane (CAS 110-54-3, 208752-1L, Sigma, US, Chromatographically pure)/chloroform (CAS 67-66-3, 288306, Sigma, US, Chromatographically pure) (4:1, *v*/*v*) was added as the organic solvent for air-dried PLFAs.

The PLFAs were identified by gas chromatography–mass spectrometry (6890 GC-5973 MS Agilent; Agilent Technologies, Santa Clara, CA, USA). The heating program was as follows: 1 min at 50 °C, 2 min at 180 °C, 2 min at 220 °C, 1 min at 240 °C, and 15 min at 260 °C. The inlet temperature was 230 °C, and the connection temperature between the gas phase and the mass spectrometer was 280 °C. The split injection mode was applied, and high-purity helium was adopted as the carrier gas with a split ratio of 10:1. The mass spectrometer used an electron ionization source at an electron energy of 70 eV.

The relative contents of each PLFA were determined based on the contents of the internal standards. Bacterial biomass was represented by the sum of PLFAs 14:0, 15:0, a15:0, i15:0, i16:0, 16:1ω5, 16:1ω7, 16:1ω9, 17:0, a17:0, i17:0, 18:0, 18:1ω7, cy17:0, and cy19:0; the quantity of 18:2ω6, 9 was used as an indicator of fungal biomass; the sum of 10Me16:0, 10Me17:0, and 10Me18:0 was used as an indicator of actinomycete biomass [35].

(2) *Biolog EcoPlate analysis*. The Biolog EcoPlate system (MicroStation, Biolog Inc., Hayward, CA, USA) used to estimate the functional diversity (metabolic potential) of soil microbial communities contains 96-well microplates with 31 different C sources and one control in three replications [36]. The C substrates can be classified into five guilds, namely, carbohydrates (CH), carboxylic and acetic acids (CA), amino acids (AC), polymers (PO), and amines and amides (AA) [37]. The C source consumption rate was indicated by the reduction of tetrazolium violet redox dye [36]. A step-by-step manual for Biolog EcoPlate analysis is provided by Sofo and Ricciuti [38].

Approximately 10 g of sieved fresh soil samples were shaken in 90 mL of sterilized pure water for 60 min at 150 rpm (30 °C). The suspension (5 mL) was diluted 100 times with sterilized pure water, and the dilution was left to stand for 5 min. Subsequently, each well of Biolog EcoPlate was continuously inoculated with 150 μL of the dilution at 25 °C for 10 d. Absorbance was measured every 24 h at a wavelength of 590 nm using a microplate reader (Biolog Microstation System version GEN III; Biolog Inc., Hayward, CA, USA). The optical density (OD_590_) value of each well was calculated by subtracting the blank control value from the value of each plate well. Microbial activity in each microplate was expressed as average well color development (AWCD) [36,38], using the following formula:

(1) $$AWCD=\sum_{i=1}^{n}\frac{\left(C_{i}-R\right)}{n}$$

where *C_i_* is the OD_590_ value in the *i*th well; *R* is the OD_590_ value of the control well; *n* is the number of C sources (*n* = 31 in this study) [39].

### 2.4. Data Analysis

We analyzed the impacts of different cultivation practices on soil chemical properties and metabolic fingerprints of soil microbial communities (represented by AWCD values at 240 h, AWCD_240_) by non-parametric comparison and principal component analysis (PCA).

(1) *Non-parametric comparison*. In each season, the AWCD values for certain C substrate groups were compared among different sample groups (US, RS, and BS). The restrictive distribution assumption on the data could not be satisfied; hence the non-parametric method (Kruskal–Wallis test) was used for multiple comparisons [40].

(2) *PCA*. As a classical multivariate statistical analysis technique, PCA identifies the principal component, a linear combination of the original variables, and displays the similarity trend across observations [41]. Both soil chemical properties and microbial C source utilization profiles were considered variables in the PCA procedure. Differences (or similarities) in the three groups of soil sampled in different seasons (e.g., BS in summer) were examined based on the selected variables, and the potential factors influencing the variables were explored. The variables were scaled to unit variance before PCA, making them comparable [42].

All data analyses were performed in R (version 4.0.2) [43] through RStudio (version 1.3.1073; https://www.rstudio.com/, accessed on 8 October 2022). The ‘PMCMRplus’ package in R [40] was employed in non-parameter comparison. PCA was executed using two R packages: ‘FactoMineR’ [44] and ‘factoextra’ (http://www.sthda.com/english/rpkgs/factoextra, accessed on 11 October 2022).

## 3. Results

### 3.1. Soil Chemical Properties

All soil samples’ pH and nutrient properties varied to some extent over the rice cultivation period (Table 1). The pH, SOM, TN, and AHN values in RS samples were generally the highest, followed by those of BS and US, although the differences were not always significant among all three groups.

**Figure 1:**
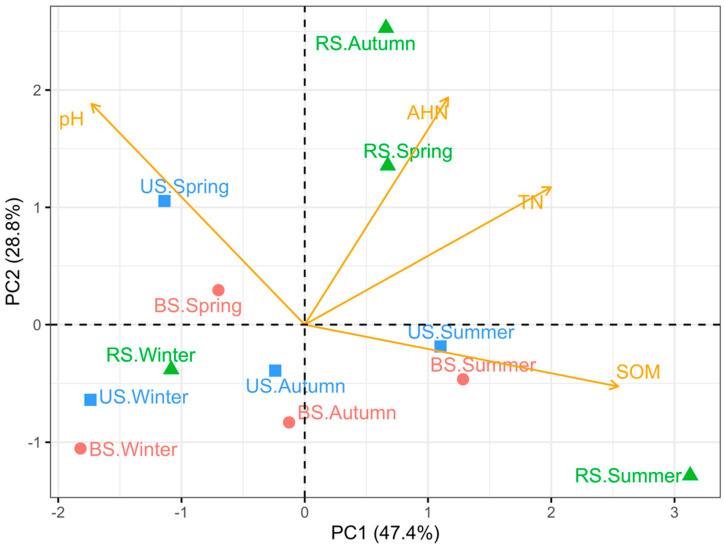
Principal component analysis (PCA) of soil chemical properties in cultivated and uncultivated land in different seasons. Rhizosphere soil had better nutrient status than bulk soil and uncultivated soil. Abbreviations: BS—bulk soil; RS—rhizosphere soil; US—uncultivated soil; SOM—soil organic matter; TN—total nitrogen; AHN—alkali-hydrolyzed nitrogen. Taking “RS.Summer” as an example, it represents the rhizosphere soil sampled in summer.

PCA plot shows that the first two principal components (PCs) accounted for 76.2% of the total variation in soil chemical properties (PC1: 47.4%; PC2: 28.8%) (Figure 1). Soil pH was significantly associated with both PC1 (*p* = 0.031) and PC2 (*p* = 0.016). SOM and TN were associated with PC1 remarkably (SOM: *p* < 0.001; TN: *p* = 0.009), whereas AHN exhibited a strong association with PC2 (*p* = 0.012).

Based on the measured chemical properties, RS samples were distinct from BS and US samples throughout the rice cultivation period, excluding winter (Figure 1). Soils collected in summer were characterized by higher SOM and TN concentrations when compared with soils sampled in winter. Compared with the BS and US samples, the RS samples had higher AHN concentrations and pH values in autumn and spring. All three groups of soils sampled in spring had high pH values. The soil chemical properties of the US and BS samples obtained in spring and winter were somewhat similar.

### 3.2. PLFA-Based Soil Microbial Biomass

**Figure 2:**
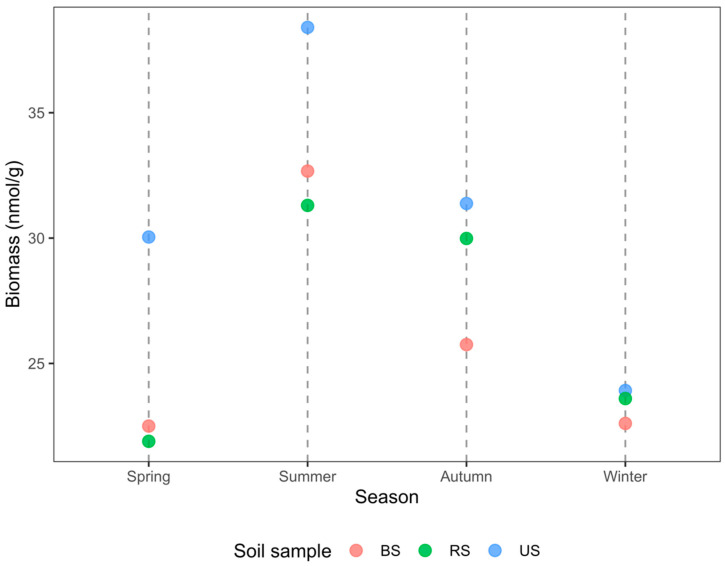
Microbial biomass measured via PLFA analysis in three groups of soil sampled in different seasons. It was consistently highest in uncultivated soil. Abbreviations: BS—bulk soil; RS—rhizosphere soil; US—uncultivated soil.

PLFAs are vital constituents of biomass in living microorganisms. According to the PLFA results, soil MB varied across seasons (Figure 2). Overall, the MB increased considerably from spring to summer and decreased continuously in autumn and winter. In both spring and summer, the total MB of BS samples was slightly higher than that of RS samples. The trend, however, was reversed during autumn and winter. Irrespective of the season, US samples had the highest MB.

### 3.3. Carbon Metabolism Characteristics of Total Carbon Sources

**Figure 3:**
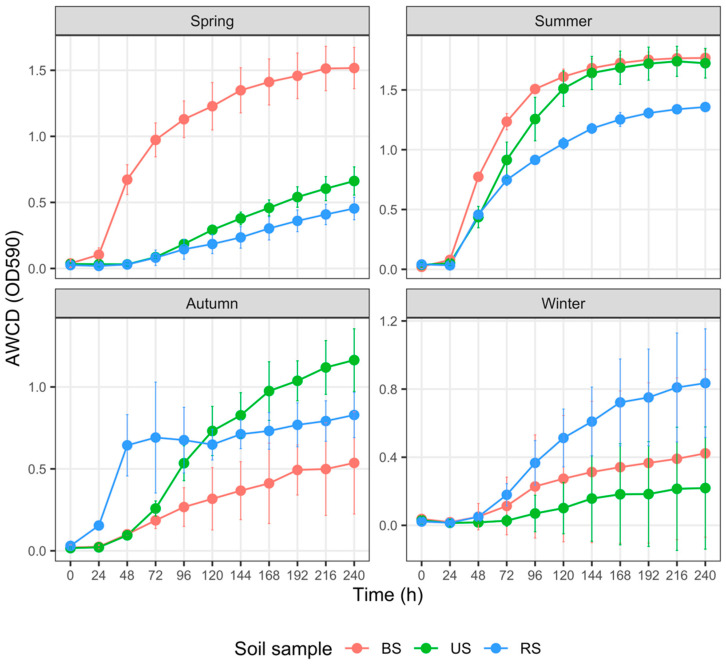
Variation in the average well color development (AWCD) of total carbon sources (*n* = 31) among three groups of soil samples collected in different seasons. The error bar represents the mean ± SD (*n* = 3). AWCD values at the 240th h for each soil sample were compared using the Kruskal–Wallis test. Abbreviations: BS—bulk soil; RS—rhizosphere soil; US—uncultivated soil.

The total carbon metabolism properties of the three soil microbial communities, represented with AWCD, were demonstrated in Figure 3. In spring, AWCD_240_ values of BS were significantly higher than those of US (*p* = 0.024) and RS (*p* < 0.001); AWCD_240_ values of US were notably higher than those of RS as well (*p* = 0.024). There was no significant difference in AWCD_240_ values between BS and US in summer (*p* = 0.768). Still, the AWCD_240_ values of RS were significantly and marginally significantly lower than those of US (*p* = 0.028) and BS (*p* = 0.067), respectively. AWCD_240_ values of US were strikingly higher than those of BS (*p* = 0.005) and marginally significantly higher than those of RS (*p* = 0.056) in autumn. In contrast, differences in AWCD_240_ values between RS and BS were not apparent (*p* = 0.175). We did not detect any remarkable differences (*p* values of all pairs were higher than 0.3) among the AWCD_240_ values of BS, RS, and US in winter.

### 3.4. Carbon Metabolism Characteristics with Five Substrate Groups

**Figure 4:**
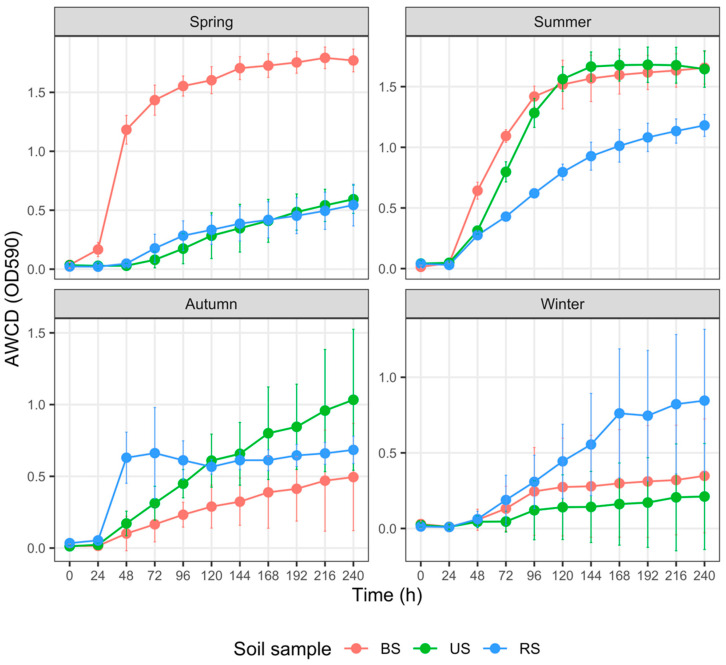
Variation in the average well color development (AWCD) of carbohydrates among three groups of soil samples collected in different seasons. The error bar represents the mean ± SD (*n* = 3). AWCD values at the 240th h for each soil sample were compared using the Kruskal–Wallis test. Abbreviations: BS—bulk soil; RS—rhizosphere soil; US—uncultivated soil.

**Figure 5:**
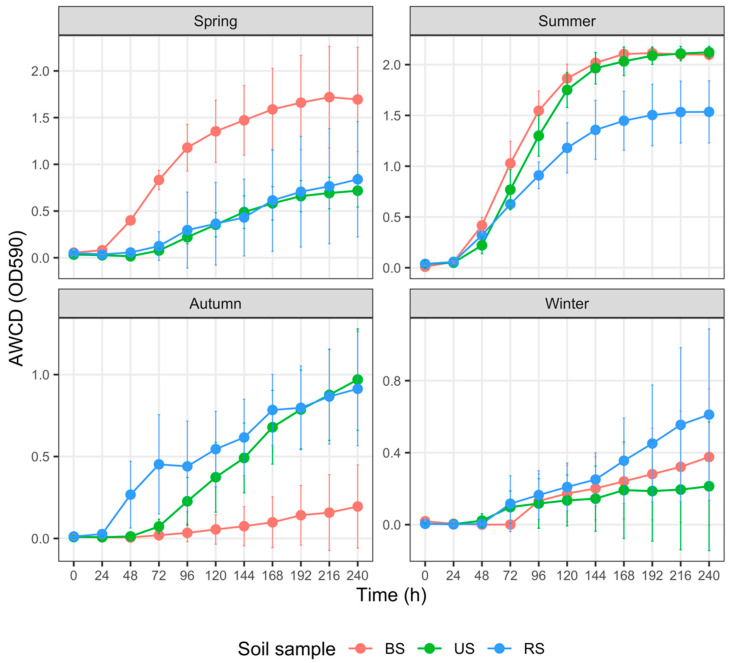
Variation in the average well color development (AWCD) of polymers among three groups of soil samples collected in different seasons. The error bar represents the mean ± SD (*n* = 3). AWCD_240_ for each soil sample was compared using the Kruskal–Wallis test. Abbreviations: BS—bulk soil; RS—rhizosphere soil; US—uncultivated soil.

**Figure 6:**
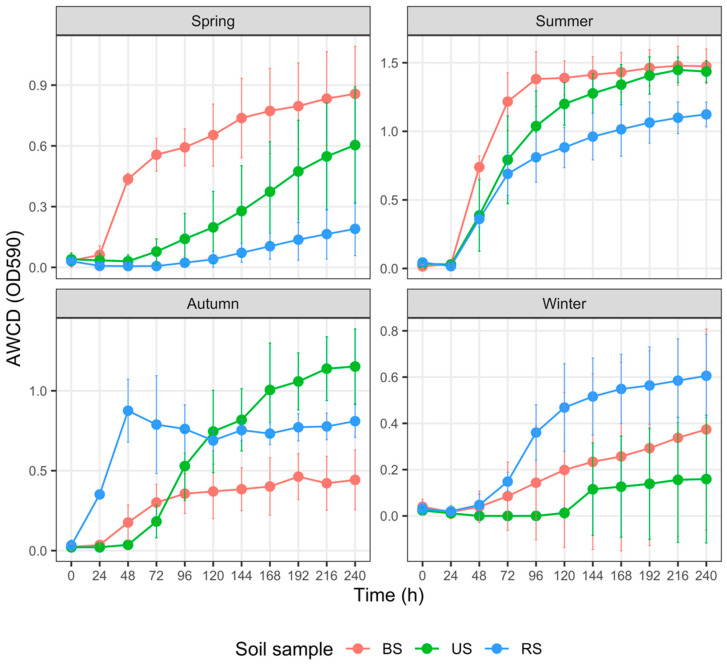
Variation in the average well color development (AWCD) of carboxylic and acetic acids among three groups of soil samples collected in different seasons. The error bar represents the mean ± SD (*n* = 3). AWCD values at the 240th h for each soil sample were compared via the Kruskal–Wallis test. Abbreviations: BS—bulk soil; RS—rhizosphere soil; US—uncultivated soil.

**Figure 7:**
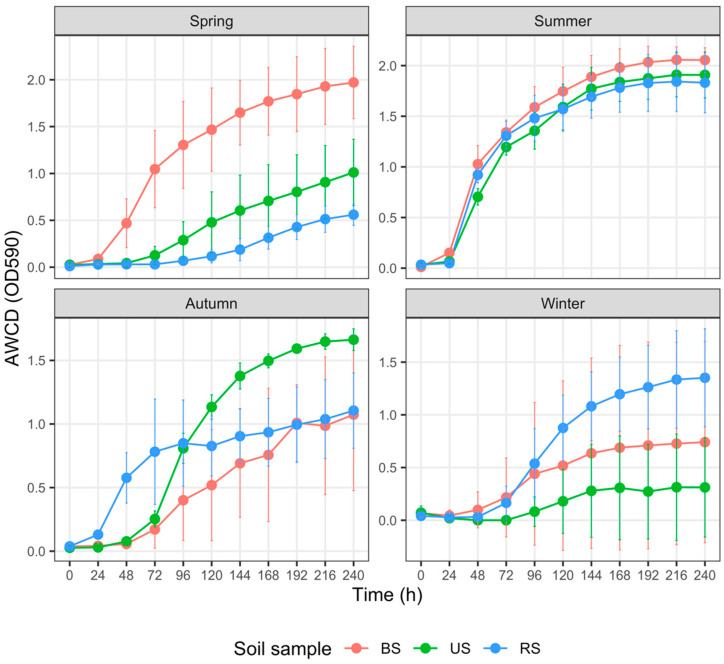
Variation in the average well color development (AWCD) of amino acids among three groups of soil samples collected in different seasons. The error bar represents the mean ± SD (*n* = 3). AWCD_240_ of each soil sample was compared using the Kruskal–Wallis test. Abbreviations: BS—bulk soil; RS—rhizosphere soil; US—uncultivated soil.

**Figure 8:**
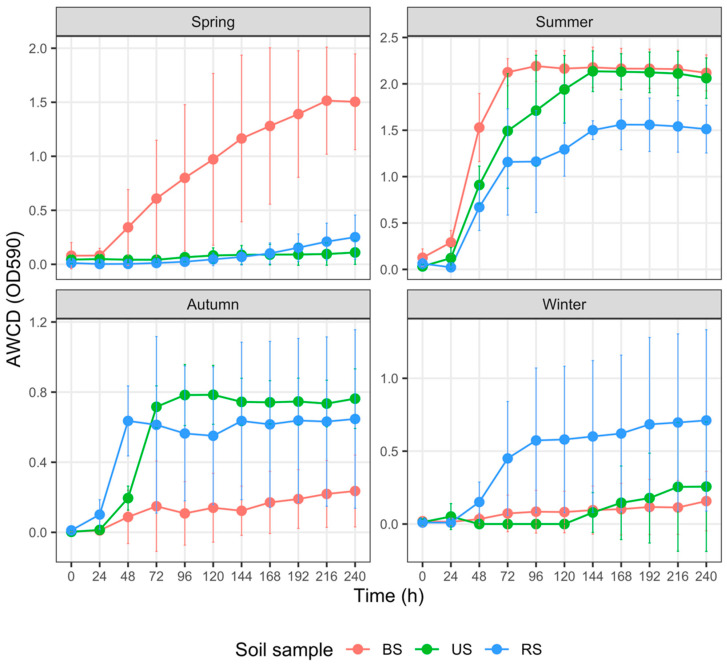
Variation in the average well color development (AWCD) of amines and amides among three groups of soil samples collected in different seasons. The error bar represents the mean ± SD (*n* = 3). AWCD_240_ for each soil sample was compared via the Kruskal–Wallis test. Abbreviations: BS—bulk soil; RS—rhizosphere soil; US—uncultivated soil.

For each group of soils sampled from spring to winter, the AWCD trends revealed varied metabolic activities on different C sources (Figure 4, Figure 5, Figure 6, Figure 7 and Figure 8). In addition, the AWCD values of each soil sample group varied seasonally.

(1) Carbohydrate utilization (Figure 4)

In spring, the AWCD_240_ values of BS samples were marginally significantly higher than those of the US samples (*p* = 0.057) and RS samples (*p* = 0.043), respectively; however, the differences between the latter two groups were not discernible (*p* = 0.972). In summer, AWCD_240_ values did not differ significantly between the BS and US samples (*p* = 0.972), both of which were notably and marginally significantly higher than those of the RS samples (*p* = 0.057 and 0.043, respectively). We observed no differences in AWCD_240_ values among the BS, RS, and US samples in autumn or winter (*p* > 0.2 for all pairs).

(2) Polymer utilization (Figure 5)

RS samples in summer had considerably lower AWCD_240_ values than the US (*p* = 0.043) and BS (*p* = 0.057) samples, whereas the latter two groups had minimal differences in AWCD_240_ values (*p* = 0.972). In autumn, the AWCD_240_ of RS samples was not significantly different from that of the US samples (*p* = 0.768), with both being higher than that of the BS samples (*p* = 0.067 and 0.028, respectively). There were minor differences in AWCD_240_ values among the three sample groups in spring and winter (*p* > 0.1 for all pairs).

(3) Carboxylic and acetic acid utilization (Figure 6)

In spring, the AWCD_240_ values of the RS samples were significantly and marginally significantly lower than those of the BS samples (*p* = 0.015) and US samples (*p* = 0.069); however, the differences in AWCD_240_ values between the US and BS samples were not considerable (*p* = 0.972). In summer, the AWCD_240_ values of the RS samples were similarly notably and marginally significantly lower than those of the BS samples (*p* = 0.043) and US samples (*p* = 0.057); however, there was no significant divergence between the latter two groups (*p* = 0.972). The US samples collected in autumn had higher AWCD_240_ values than the BS samples (*p* < 0.001) and RS samples (*p* = 0.020), whereas the AWCD_240_ values of the RS samples were strikingly higher than those of the BS samples (*p* = 0.020). The differences in AWCD_240_ values among the BS, RS, and US samples diminished in winter (*p* > 0.2 for all pairs).

(4) Amino acid utilization (Figure 7)

In spring, the RS samples had remarkably lower AWCD_240_ values than the US samples (*p* = 0.024) and BS samples (*p* ≤ 0.001), whereas the AWCD_240_ values of the BS samples were significantly higher than those of the US samples (*p* = 0.024). In autumn, the AWCD_240_ values of the US samples were higher than those of the BS samples (*p* = 0.043) and RS samples (*p* = 0.057); nevertheless, the differences in AWCD_240_ between BS and RS samples were not remarkable (*p* = 0.972). There were minor differences inAWCD_240_ among the three sample groups in summer and winter (*p* > 0.3 for all pairs).

(5) Amines and amide utilization (Figure 8)

The AWCD_240_ values of the BS samples in spring were marginally significantly and significantly higher than those of the RS samples (*p* = 0.067) and US samples (*p* = 0.028); however, the differences in AWCD_240_ between the US and RS samples were minimal (*p* = 0.768). In summer, the AWCD_240_ values of the RS samples were notably and marginally significantly lower than those of the BS (*p* = 0.028) and US samples (*p* = 0.067), although there was no significant divergence in AWCD_240_ between the US and BS samples (*p* = 0.768). We observed no apparent differences in AWCD_240_ among the BS, RS, and US samples in autumn and winter (*p* > 0.1 for all pairs).

(6) PCA of carbon source utilization profiles

**Figure 9:**
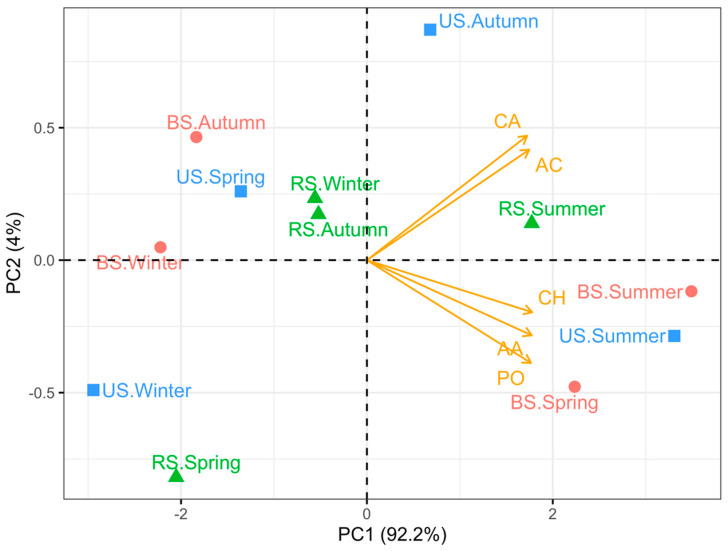
Principal component analysis (PCA) of carbon source utilization profiles of soil microorganisms in cultivated and uncultivated land. There was an apparent difference in carbon source utilization patterns for soil microbiomes in each season. Abbreviations: BS—bulk soil; RS—rhizosphere soil; US—uncultivated soil; CH—carbohydrates; AA—amines and amides; PO—polymers; AC—amino acids; CA—carboxylic and acetic acids. Taking “RS.Summer” as an example, it represents the rhizosphere soil sampled in summer.

According to the PCA results, the first two principal components explained 96.2% of the total variation in CMC (PC1: 92.2%; PC2: 4%; Figure 9). All the major C substrate guilds were strikingly associated with PC1 (*p* < 0.001) in the following order: CH > AA > PO > AC > CA. US samples collected in different periods preferred distinct C sources from the microbial metabolic activities of specific C substrates. BS samples collected in spring and summer exhibited a preference for similar C substrate guilds, which was different from that of BS samples in other seasons. RS samples in winter and autumn were more similar in the C source utilization patterns than the RS samples in either spring or summer.

## 4. Discussion

### 4.1. Rhizosphere Soil Possesses Superior Chemical Properties

Consistent with previous reports [8,45], conventional rice monoculture practices in our experimental field induced drastic changes in soil pH and nutrient concentrations. The maximum values of soil chemical properties were observed in the RS samples in most cases (Table 1), which somewhat contradicts our first hypothesis that rice cropping activities negatively impact nutrient-related soil chemical properties.

The rhizosphere, defined as the soil contiguous to the plant roots [46], is a crucial zone modulating the C and N biogeochemical cycles [47]. Roots release various organic and inorganic substances, including amino acids, sugars, organic acids, and mineral nutrients, into the surrounding soil during plant growth [46,48,49]. Therefore, the rhizodeposition process may be responsible for the higher concentrations of SOM and N nutrients in RS samples in the present study.

It is estimated that 50% of the C fixed by photosynthesis enters the soil through rhizodeposition [48], naturally elevating SOM within rhizosphere soil immediately. This mechanism is broadly consistent with our findings in the cultivated plot. Moreover, soils sampled in the rapid growth stage (summer) and fallow stage (winter) of paddy rice were distributed at both ends along the PC1 axis, and PC1 was most prominently associated with SOM concentration (Figure 2), implying a shift in rhizosphere C deposition of paddy rice across different growth stages [48,50]. Similarly, Lu et al. [51] reported that the proportion of photosynthetic C of rice allocated underground decreased drastically from 28% to 2% from tillering to maturity.

In addition to SOM, soil N compounds are critical components for soil fertility in agricultural land. N deficiency and/or an inappropriate ratio of carbon to nitrogen inhibits microbial activity and severely limits plant growth [52]. Although N derived from rhizodeposition is less considered than organic C compounds [49], it can presumably be used to explain our observation that the TN concentration was relatively high in the RS samples (Table 1). Tremendous amounts of N are potentially released into the soil as rhizodeposits, accounting for 71% of total assimilated plant N and 96% of total belowground plant N [49]. In addition, N rhizodeposition can vary across plant growth stages [53]. A previous study found that the concentrations of soil N derived from rhizodeposits of spring wheat increased over the plant growth period [54]. This is consistent with the steadily increasing TN concentration in the RS samples (Table 1) and the apparent divergence in TN concentration of RS samples plotted along PC1, which was significantly associated with TN concentration (Figure 1).

AHN, also known as available N, can be the limiting factor for plant growth and productivity [55]. Environmental temperature and plant growth stage, which influence soil microbial and biochemical processes and N uptake efficiency, are the major factors controlling soil N availability [55,56]. In the present study, soil AHN concentration exhibited a decreasing trend from spring to winter, corresponding to different rice growth stages (Figure 1). The highest AHN concentrations within the spring soils can be attributed to N fertilizer application and a slower growth rate of rice seedlings. The subsequent variation in AHN is similar to trends reported in other studies focusing on the temporal dynamics of soil available N in various ecosystems [55,56,57]. Furthermore, there was a visible discrepancy in AHN concentration in RS samples when compared with the other two groups in autumn (Figure 1), implying intense microbial activity related to N cycling within the rhizosphere soil [8,46].

### 4.2. Rice Cultivation Reduces Soil Microbial Biomass

Microorganisms execute critical functions and modulate soil physicochemical properties, e.g., structure, porosity, fertility, and availability of limited nutrients [20,58]. In addition, MB is involved in residue decomposition and nutrient cycling [59], thereby contributing to ecosystem sustainability. MB is frequently utilized to illustrate the responses of soil microbiota to changing environmental conditions [59,60,61]. MB was comparatively higher in the uncultivated plot (US samples) than in the cultivated plot (BS and RS samples), especially in the rice growth period (Figure 2). Agricultural intensification, for example, in the forms of tillage, fertilization, and monoculture, leads to declines in soil biological properties and health [7,62].

Zero-tillage soil has macro-porosity and better connectivity [63]; however, conventional tillage practices could inhibit the mineralization and migration of nutrients and reduce microbial activity and biomass [64,65], either directly or indirectly [66]. Mathew et al. [12] explored differences in soil microbiological properties between tilled and untilled corn systems. They observed that soils under long-term no-till retained higher MB because of favorable physicochemical conditions for microbial activity [12]. In addition, chemical fertilizer application has been demonstrated to reduce soil MB [67]. The impacts probably arise from shifts in soil microbial community function, i.e., the transformation toward a more *r*-selected microbial community [10,68,69]. Such community shifts potentially arise directly from increased nutrient availability and indirectly through variations in soil C, pH, or other associated properties [69,70]. Furthermore, Xuan et al. [19] examined differences in soil bacterial communities between crop rotation fields and intensive rice cultivation. They observed that bacterial community composition, abundance, and diversity in the rotation system were prominently distinct, with higher values than in the rice monoculture system [19].

### 4.3. Monoculture Practices Influence Soil Carbon Metabolism Characteristics

Two standard cultivation practices, i.e., tillage and fertilization, modulate factors influencing the soil microbial community-level physiological profiles [71,72,73]. AWCD, acquired using the Biolog Ecoplate system, enables the portrayal of the metabolic fingerprints of soil microbial communities and distinguishing the metabolic activity of soil microorganisms across treatments [74,75].

Numerous factors, such as fertilizer type and rate of application, soil properties, and crop species, regulate soil microbial responses to fertilization [72]. In the cultivated plot, N, P, and K fertilization in spring prominently increased the AWCD values of BS samples (Figure 3, Figure 4, Figure 7 and Figure 8). Higher AWCD values generally imply higher metabolic functional diversity and activity [74,75]. In other words, after a long winter-fallow period, fertilizer amendment in the cultivated plot could strongly stimulate the growth of soil microorganisms, which is intuitively attributed to the nutrient influx of fertilizer. In contrast to the AWCD trend, however, the MB in BS samples at the same period was lower than that in the US samples (Figure 2), denoting that different soil microbial properties are influenced unequally by fertilization [69,72].

Compared with tillage, no-till management has been shown to increase soil microbial functional diversity [4,12,76]. In the present study, the negative impact of tillage treatment on AWCD emerged in the reproductive phase of rice (summer) and became significant until the harvesting phase (autumn; Figure 5), leading to a remarkable decline in the metabolic functional diversity within cultivated soils during the vital stages of rice production. Such a pattern might be explained by the fading impact of fertilization and the adverse impact of tillage. Tillage practices, such as plowing, disrupt soil aggregates, alter soil structure, and decrease soil stability, reducing capillary pore space and destroying microhabitats of soil microorganisms [69,72]. Such soil environmental changes are detrimental to microbial activity; for instance, tilled soil exhibits poorer dehydrogenase activity, an indicator of the overall microbial activity and oxidation–reduction reactions in soil [76].

Rice management strategies, however, are just one factor impacting the soil microbial carbon metabolism properties. In a comparable study performed by Zhao et al. in Changchun (Northeast China) [77], the variation patterns of AWCD values and differences among AWCD_240_ values of three soil samples at each season (Supplementary Materials Figure S1) were not entirely consistent with our results (Figure 3), albeit the soil types, cultivation measures, and soil MB properties were similar to some extent [77,78]. It is indicated that local environmental factors, including temperature, light conditions, soil grain size, and nutrient status, would also determine the microbial community composition and dynamics. Therefore, a comprehensive experiment harboring more potential parameters should be performed in the future to evaluate the relative contribution of a variety of factors.

Soil microbial functional responses to cultivation practices may vary seasonally [4,10,76]. Throughout the rice growth period, soil microorganisms under different cultivation practices exhibited inconsistent preferences for the five groups of C substrates. However, there is a relatively discernible difference in C substrate preference between summer and the other seasons (Figure 9). Studies have shown that higher temperature and adequate moisture in summer could trigger soil microbial growth, activity, and community diversity [60,79], which are consistent with our findings to some extent.

The PLFA analysis and Biolog EcoPlate assays are traditional measures disclosing the soil microbial community’s biomass, composition, and physiological profile [20]. However, taxonomic information and the function of a given microbial community cannot be characterized using these tools alone. Therefore, molecular biology approaches, such as rRNA methods, will fill the gap in understanding the responses of soil microbes under diverse cultivation practices.

## 5. Conclusions

It has been confirmed that soil quality deteriorates over long-term continuous rice monocultivation. Based on our results, (1) such practices indeed altered soil pH and nutrient concentrations; however, the chemical properties of the rhizosphere soil were superior, somewhat contradicting the first hypothesis. (2) Soil MB was reduced due to agricultural activities, such as fertilization, tillage, and monoculture, supporting the second hypothesis, although the adverse impacts varied by season. (3) Coinciding with the last hypothesis, microbial CMC, measured by the AWCD value, changed under cultivation and exhibited seasonal variation.

Herein, we emphasize that specific cultivation practices may elicit distinct responses from soil microbial communities based on total biomass and C source utilization profiles. Despite no consistent adverse responses of soil chemical properties under rice cultivation, the microbial properties seemingly respond clearly and sensitively. Accordingly, soil microbial properties could be considered superior indicators in reflecting soil disturbance. Genomic-based approaches will help further deepen the insight into the phylogenetic and functional dynamics of the microbial communities in degraded soils.

## Figures and Tables

**Table 1:** Chemical properties of soil samples from cultivated and uncultivated plots in different seasons. In most cases, soil nutrient indicators (SOM, TN, and AHN) were higher in rhizosphere soil.

Seasons	Samples	pH	SOM (g/kg)	TN (g/kg)	AHN (mg/kg)
Spring	RS	6.3 ± 0.1	33.6 ± 3.0	1.45 ± 0.4	197 ± 15
	BS	6.2 ± 0.1	25.8 ± 3.0	1.28 ± 0.1	157 ± 12
	US	6.4 ± 0.2	26.6 ± 3.0	1.24 ± 0.2	162 ± 13
Summer	RS	5.8 ± 0.2	41.1 ± 4.0	1.86 ± 0.3	145 ± 12
	BS	6.1 ± 0.1	36.2 ± 2.0	1.66 ± 0.4	133 ± 12
	US	6.2 ± 0.3	36.3 ± 2.0	1.77 ± 0.2	122 ± 11
Autumn	RS	6.5 ± 0.1	29.5 ± 3.0	2.38 ± 0.2	157 ± 13
	BS	6.2 ± 0.2	31.6 ± 2.0	1.53 ± 0.3	94.2 ± 10
	US	6.3 ± 0.1	32.2 ± 1.0	1.56 ± 0.2	97.2 ± 11
Winter	RS	6.4 ± 0.1	31.6 ± 3.0	1.23 ± 0.2	97 ± 9
	BS	6.2 ± 0.2	23.4 ± 1.0	1.14 ± 0.1	91 ± 8
	US	6.3 ± 0.1	25.2 ± 3.0	1.20 ± 0.2	93 ± 9

Abbreviations: BS—bulk soil; RS—rhizosphere soil; US—uncultivated soil; SOM—soil organic matter; TN—total nitrogen; and AHN—alkali-hydrolyzed nitrogen.

## Data Availability

Not applicable.

## Supplementary Materials

The following supporting information can be downloaded at: https://www.mdpi.com/article/10.3390/microorganisms10112153/s1, Figure S1. Variation in the average well color development (AWCD) of total carbon sources (*n* = 31) among three groups of soil samples collected in different seasons in Zhao’s research.
