# Supplementary material for: Unraveling the Impact of Long-Term Rice Monoculture Practice on Soil Fertility in a Rice-Planting Meadow Soil: A Perspective from Microbial Biomass and Carbon Metabolic Rate
Source: Microorganisms. 2022 Oct 30;10(11):2153. doi: 10.3390/microorganisms10112153 (PMC9699473; doi:10.3390/microorganisms10112153)
Supplement: Supplementary file 1 [file microorganisms-10-02153-s001.zip › microorganisms-1905911-supplementary.pdf]

Zhao et al. conducted a comparable study in an agricultural technology extension station in Changchun [1], located ca. 190 km southwest of our experimental field. Both soils of the two experiment fields belonged to the black soil groups [2]. Additionally, they underwent similar rice cultivation strategies and presented similar soil microbial biomass patterns.

In Zhao's study, the total carbon metabolism properties of the microbial communities within bulk soils, rhizosphere soils, and uncultivated soils, represented with AWCD, were presented in Figure S1. In spring and winter, no significant differences (p values of all pairs were higher than 0.1) were detected among the $AWCD_{240}$ values of BS, RS, and US. There was no apparent difference in $AWCD_{240}$ values between BS and RS in summer (p=0.768); however, the $AWCD_{240}$ values of US were significantly and marginally significantly higher than those of BS (p=0.028) and RS (p=0.067), respectively. $AWCD_{240}$ values of US were strikingly higher than those of RS (p=0.028) and marginally significantly higher than those of BS (p=0.067) in autumn. In contrast, differences in $AWCD_{240}$ values between RS and BS were not remarkable (p=0.768).

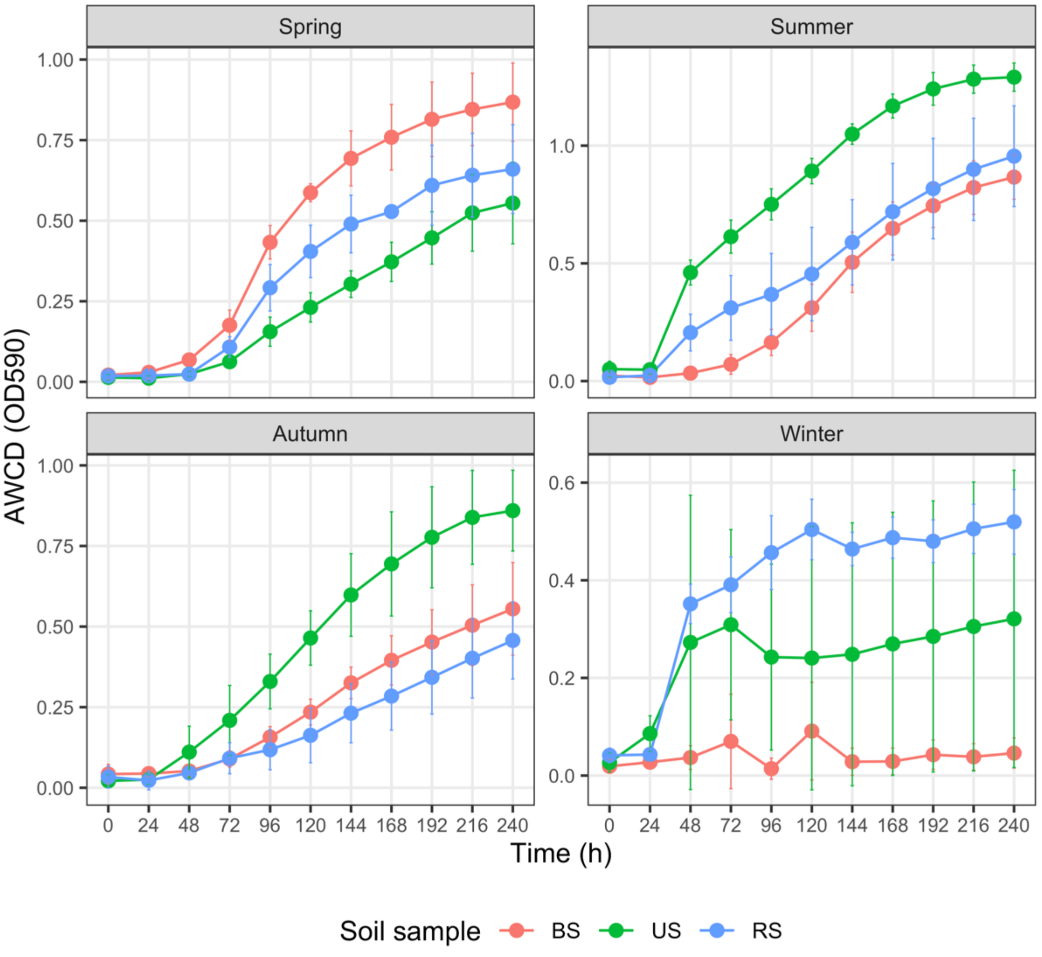

Figure S1. Variation in the average well color development (AWCD) of total carbon sources (n = 31) among three groups of soil samples collected in different seasons in Zhao's research. The error bar represented the mean ± SD (n = 3). AWCD values at the 240th h for each soil sample were compared using the Kruskal-Wallis test. Abbreviations: BS - bulk soil; RS - Rhizosphere soil; US - uncultivated soil. We redrew this figure based on information from Zhao *et al.* [1].

References

1. Zhao, Z.; Cui, B.; Hou, Y.; Liu, S.; Wang, Y. Impact on the Microbial Biomass and Metabolic Function of Carbon Source by Black Soil during Rice Cultivation. *Environmental Science* **2015**, *36*, 3011–3017.
2. Dou, S.; Guo, D. Soil Type Distribution and Black Soil Land Protection in Jilin Province. *Journal of Jilin Agricultural University* **2018**, *40*, 449–456.
